# IMPROVING VIDEO DEVICE USAGE AMONG OLDER RURAL VETERANS WITH USER-CENTERED DESIGN

**DOI:** 10.1093/geroni/igac059.2235

**Published:** 2022-12-20

**Authors:** Hillary Lum, Lynette Kelley, Kathryn Nearing, Leah Walker, Gerhardt Johnson, Steven Barczi

**Affiliations:** University of Colorado Anschutz Medical Campus, Aurora, Colorado, United States; Department of Veteran Affairs, Denver, Colorado, United States; VA Eastern Colorado Geriatric Research Education and Clinical Center, Denver, Colorado, United States; Department of Veterans Affairs, Madison, Wisconsin, United States; Department of Veterans Affairs, Madison, Wisconsin, United States; Department of Veterans Affairs, Madison, Wisconsin, United States

## Abstract

The Veterans Administration (VA) established pathways to provide VA-issued tablets and increased access to internet for Veterans without these resources. Veterans aged above 65, experience barriers with telemedicine such as access and usability. We sought to improve the usability and experience of telemedicine for rural, older Veterans receiving a VA-issued tablet by modifying materials and qualitatively evaluating their experience with set-up and preparing for their first appointment, guided by user-centered design. We conducted a rapid exploratory evaluation, to understand Veteran and care partner experiences setting up VA-issued tablets and logging into their first appointment using standard 9-page instructions. We interviewed telehealth technicians, and providers to better understand patient barriers from their perspective. Using insights from interviews and evidence-informed guidelines on educational materials for older adults, we created a two-page guidance. A group of Veterans and care partners reviewed the materials and provided feedback. Received feedback provided 17 suggestions, 8 of which were utilized, including enlarging graphics, clarifying abbreviations (or wording), consolidating the instructions further and emphasizing pertinent information further. Modified materials reduced standard written instructions from nine pages to two. Feedback suggests that updated materials are helpful, aesthetically pleasing and preferred over current materials. Utilizing user-centered design methods, addressed barriers experienced by older, rural Veterans with initial telemedicine device and appointment set-up. Veterans, care partners, providers, and telehealth technicians perceived materials adapted for older adults as supportive of video device usability, helping to alleviate barriers that prevent Veterans from initiating telemedicine.

